# RETRACTION: Investigating How Tamsulosin Combined With Levofloxacin Impacts Wound Healing in Patients With Chronic Prostatitis who May also Have Perineal or Urethral Wounds

**DOI:** 10.1111/iwj.70529

**Published:** 2025-04-18

## Abstract

**RETRACTION:** YangJ. , BaoC. , and GuJ. , “Investigating How Tamsulosin Combined With Levofloxacin Impacts Wound Healing in Patients With Chronic Prostatitis who May also Have Perineal or Urethral Wounds,” International Wound Journal 21, no. 1 (2024): e14656, 10.1111/iwj.14656.38272823 PMC10789924

The above article, published online on 15 January 2024, in Wiley Online Library (http://onlinelibrary.wiley.com/), has been retracted by agreement between the journal Editor in Chief, Professor Keith Harding; and John Wiley & Sons Ltd. Following an investigation by the publisher, all parties have concluded that this article was accepted solely on the basis of a compromised peer review process. The editors have therefore decided to retract the article. The authors did not respond to our notice regarding the retraction.

**RETRACTION:** J. Yang , C. Bao , and J. Gu , “Investigating How Tamsulosin Combined With Levofloxacin Impacts Wound Healing in Patients With Chronic Prostatitis who May also Have Perineal or Urethral Wounds,” International Wound Journal 21, no. 1 (2024): e14656, 10.1111/iwj.14656.38272823 PMC10789924

The above article, published online on 15 January 2024, in Wiley Online Library (http://onlinelibrary.wiley.com/), has been retracted by agreement between the journal Editor in Chief, Professor Keith Harding; and John Wiley & Sons Ltd. Following an investigation by the publisher, all parties have concluded that this article was accepted solely on the basis of a compromised peer review process. The editors have therefore decided to retract the article. The authors did not respond to our notice regarding the retraction.

